# The perinatal factors that influence the excretion of fecal calprotectin in premature-born children

**DOI:** 10.1515/med-2022-0522

**Published:** 2022-07-13

**Authors:** Jelena R. Cekovic, Nikola S. Prodanovic, Sara S. Mijailovic, Sanja M. Knezevic, Biljana P. Vuletic, Andjelka K. Stojkovic, Dragana M. Savic, Tijana V. Prodanovic, Marina M. Stanojevic, Aleksandra M. Simovic

**Affiliations:** Neonatal Intensive Care Unit, Center for Neonatology, Pediatric Clinic, University Clinical Centre Kragujevac, Kragujevac, Serbia; Department of Alloartoplastic Surgery, Clinic for Orthopedics and Traumatology, University Clinical Center Kragujevac, 34000 Kragujevac, Serbia; Department of Surgery, Faculty of Medical Sciences, University of Kragujevac, Svetozara Markovica 69, 34000 Kragujevac, Serbia; Department of Medical Statistics and Informatics, Faculty of Medical Sciences, University of Kragujevac, Kragujevac, Serbia; Department of Pediatrics, Faculty of Medical Sciences, University of Kragujevac, Kragujevac, Serbia; Department of Neonatology, Clinic for Gynecology and Obstetrics, University Clinical Centre Kragujevac, Kragujevac, Serbia; Department of Cardiology, Pediatric Clinic, University Clinical Centre Kragujevac, Kragujevac, Serbia; Department of Gastroenterology, Pediatric Clinic, University Clinical Centre Kragujevac, Kragujevac, Serbia; Department of Pulmonology, Pediatric Clinic, University Clinical Centre Kragujevac, Kragujevac, Serbia

**Keywords:** preterm newborn, calprotectin, necrotizing enterocolitis

## Abstract

This study aimed to provide additional information on the influence of perinatal factors on fecal (f)-calprotectin values in preterm infants. Calprotectin was determined from the first spontaneous stool (analyzed on the Alegria device by using the enzyme-linked immunosorbent assay [ELISA] method) obtained from neonates at a mean age of 3.41 ± 2.44 days of life. We analyzed 114 subjects who had a body weight of 1847.67 ± 418.6 g and were born at a gestational age of 32.6 ± 2.43 weeks, without intestinal and other congenital anomalies or any diseases other than those related to premature birth. The values of f-calprotectin are in a positive correlation with female subjects, intrauterine growth restriction, significant ductus arteriosus, enteral feeding intolerance, postnatal prolonged use of broad-spectrum antibiotics, and values ​​of bicarbonates (analyzed in a sample of capillary arterial blood). Measurement of f-calprotectin in the first 7 days after birth can help to early detect the intestinal distress or early staging of necrotizing enterocolitis in premature infants.

## 1 Introduction

Calprotectin is the main component of soluble cytosolic proteins in human neutrophil granulocytes. The granulocytes excrete calprotectin actively into the lumen of the digestive tract [1,2,3,4,5]. Calprotectin concentration in the stool is about six times higher than in the plasma [6]. During the neonatal period, the levels of fecal (f)-calprotectin have been observed to be significantly higher, in both term and preterm infants, compared to the reference values ​​established for children and adults [1,2,3]. There is growing evidence of the potential role of calprotectin as a non-invasive diagnostic screening test of inflammation and the influence of stress factors on the digestive tract of the newborns, such as respiratory distress syndrome (RDS), perinatal asphyxia, significant ductus arteriosus, and the influence of some drugs (e.g., indomethacin or ibuprofen) [1,3]. It is of interest to determine the interdependence of f-calprotectin and the method of delivery, prenatal (gestational) or postnatal age, milk diet, volume of enteral feeding, enteral nutrition tolerance, and intestinal microflora [4,5,7,8,9]. All of the aforementioned factors, especially the quality of enteral colonization, are seen as possible modulating factors of the activity and differentiation of immune cells [7,8,10,11,12]. Some authors believe that bifidobacteria supplementation in the neonatal period may be associated with a significant reduction in f-calprotectin levels [7,8,10,11,12,13]. This study was aimed to provide more information regarding the factors that influence the excretion of f-calprotectin in premature-born children.

## 2 Materials and methods

The sample size was calculated “a priori” (G-power software), based on the following baseline parameters: study power (80%), clinically significant value of Pearson’s correlation coefficient (0.35) and first type error (*α*) (0.05). The required sample size in the study group is at least 61 subjects.

### 2.1 Ethics statement

This study was approved by the ethics committee of the University Clinical Center Kragujevac, Serbia 01.19.1973 13.05.2019 and Faculty of Medical Sciences, University of Kragujevac, Serbia. Data were prospectively collected over a period from January 2019 to September 2021.

The subjects included in our study were 114 premature infants (61 males and 53 females) hospitalized at the Neonatal Intensive Care Unit (NICU) of the Center for Neonatology, the University Clinical Center Kragujevac, Serbia. The inclusion criteria were as follows: gestational age of <37 weeks, postnatal age of <7 days, and the absence of any disease other than those related to premature birth (hyaline membrane disease, perinatal asphyxia, patent ductus arteriosus, anemia, etc.). Excluding criteria were death in the first 24 h of life, gestational age of <24 weeks, postnatal age of ≥7 days at the time of stool collection, gastrointestinal and other congenital anomalies, chromosomal aberrations, and congenital metabolic diseases.

After admission to the NICU, all the preterm infants were fed a reconstituted formula for preterm infants, as they were transported from 12 distant maternity hospitals. Calprotectin was determined from the first spontaneous thick, greenish-black and sticky stools after birth. Stools were collected once a day until 10 am from the infants at a mean age of 3.41 ± 2.44 days of life. Stool samples were not stored in the refrigerator. The duration of meconium passage was significantly prolonged in infants with very preterm (28–32 weeks), and especially, extremely preterm birth (less than 28 weeks). The values of f-calprotectin were analyzed immediately after taking the stool samples to the laboratory to be tested on the Alegria device, (24 Alegria^®^ strips, range 0–1,000 µg/g) performing the enzyme-linked immunosorbent assay (ELISA) [14,15]. Blood was taken for laboratory processing at the same time as the stool was collected.

During the study period, all preterm infants underwent echocardiography on the third day of life. Significant ductus arteriosus was defined [16,17,18,19] as a ductal diameter >1.5 mm and the presence of at least 2 of the following criteria: left atrium/aorta ratio ≥1.6, pulsatile flow in the arterial duct, retrograde or absent diastolic flow in the anterior cerebral artery or descending aorta, or fractional shortening <40%.

### 2.2 Statistical analysis

Statistical analyses were performed using SPSS version 18.0 (SPSS, Chicago, IL, USA). The continuous variables were presented as the mean value and standard deviation (SD) or median and interquartile range (IQR). The categorical data were presented as the number and proportion (%). Comparisons of f-calprotectin according to clinical factors were performed by Mann–Whitney *U* test. Spearman’s rank correlation coefficient was used for correlating f-calprotectin and continuous variables. A value of *p* < 0.05 was considered statistically significant.

## 3 Results

The demographic characteristics of the patients included in the study are shown in Table 1 and other clinical and laboratory variables included in the investigation are shown in Tables 2 and 3. In our study, the median level of fecal calprotectin in the first 7 days of life was 76.12 ± 175.44 μg/g. Female subjects had significantly higher levels of f-calprotectin, while birth weight, gestational age, delivery mode, and a 5-minute Apgar score did not significantly affect its values (Table 1).

**Table 1:** Association of demographic characteristics of patients and calprotectin

Characteristic (*n* = 114)	Mean value ± SD; median (IQR)	*p*
Gender		0.003*
Male (*n* = 61)	66.57 ± 184.15; 18.3 (31.7)	
Female (*n* = 53)	86.93 ± 166.10; 30.0 (63.0)	
Birth weight (g)	1847.67 ± 418.60; 1830.0 (567.5)	0.351^**^
Gestational age (weeks)	32.60 ± 2.43; 33.0 (4.0)	0.445^**^
Apgar score	6.53 ± 1.65; 7.0 (2.0)	0.735^**^
Delivery mode		0.571^*^
Vaginal (*n* = 50)	70.28 ± 157.64; 23.7 (39.9)	
Caesarean section (*n* = 64)	80.75 ± 189.49; 23.4 (37.4)	

SD = standard deviation; IQR = interquartile range; *Mann–Whitney *U* test; ^**^ Spearman correlation.

**Table 2:** Correlations of laboratory values and calprotectin

Variables	Mean value ± SD	Median (IQR)	Normative value*	Spearman *ρ*/*p* value
Fecal calprotectin (µg/g)	76.12 ± 175.44	23.0 (36.60)		—
CRP (mg/L)	14.97 ± 31.93	2.95 (7.43)	0.0–7.0	–0.020/0.831
Serum procalcitonin (ng/mL)	11.55 ± 18.96	4.19 (13.71)	0.5–2.0	0.008/0.935
Urea (mmol/L)	3.83 ± 2.53	3.2 (3.90)	1.1–9.1	0.019/0.846
Creatinine (µmol/L)	71.37 ± 19.28	69.5 (23.75)	49.0–106.0	0.015/0.878
pH value	7.29 ± 0.11	7.3 (0.15)	7.30 –7.45	0.020/0.830
pCO2 (kPa)	6.80 ± 2.23	6.30 (3.10)	3.5–4.5	0.052/0.583
pO2 (kPa)	6.10 ± 1.92	5.95 (2.20)	8.0–10.0	–0.037/0.695
Sodium (mmol/L)	133.57 ± 4.25	133.5 (5.0)	133.0–146.0	–0.084/0.378
Potassium (mmol/L)	5.70 ± 1.42	5.6 (1.90)	4.6–6.7	–0.040/0.678
Calcium (mmol/L)	1.18 ± 0.15	1.20 (0.19)	1.04–1.52	0.011/0.910
Glycaemia (mmol/L)	4.81 ± 4.04	3.80 (2.80)	1.5–5.5	0.076/0.424
Bicarbonates (mmol/L)	22.74 ± 4.18	22.60 (4.7)	22.0–28.0	**0.215/0.022**
Base excess (mmol/L)	–2.72 ± 4.35	–3.00 (5.2)	–1.0 to 1.0	0.039/0.685
Leukocytes (×10^9^/L)	15.62 ± 8.73	13.71 (8.6)	5.0–21.0	0.060/0.526
Neutrophils (%)	48.75 ± 12.28	49.50 (17.7)	55.0–65.0	0.168/0.080
Erythrocyte (×10^12^/L)	4.73 ± 0.90	4.80 (1.31)	4.22–5.95	–0.029/0.759
Hemoglobin (g/L)	173.40 ± 32.74	176.25 (49.3)	179.0–209.0	–0.047/0.619
Hematocrit (L/L)	0.60 ± 0.87	0.52 (0.15)	0.59–0.71	–0.062/0.523
Thrombocytes (×10^9^/L)	216.13 ± 114.68	191.45 (130.8)	150.0–350.0	–0.083/0.382

*Normal laboratory values for newborns <37 weeks of gestation, up to 7 days of age. https://www.bettersafercare.vic.gov.au/clinical-guidance/neonatal/normal-laboratory-values-for-neonates.

**Table 3:** Association of laboratory values and calprotectin

	*N* (%)	Median (IQR)	*p*
RDS			
Yes	82 (71.9)	23.4 (34.9)	
No	32 (28.1)	25.3 (44.4)	0.794^*^
Asphyxia			
Yes	58 (50.9)	20.3 (27.1)	
No	49 (49.1)	29.5 (50.5)	0.124^*^
Meal volume	—	45.5 (25.5)	0.916^**^
Intrauterine growth retardation			
Yes	10 (8.8)	26.7 (39.5)	
No	104 (91.1)	15.5 (9.2)	**0.039** ^*^
Vomiting			
Yes	34 (29.8)	35.9 (70.3)	
No	80 (70.2)	20.2 (30.2)	**0.038** ^*^
Silverman score	—	4.0 (3.0)	0.842^**^
Mechanical ventilation			
Yes	45 (39.5)	26.7 (42.9)	
No	69 (60.5)	23.4 (34.1)	0.993^*^
Dopamine			
Yes	9 (7.9)	17.3 (21.8)	
No	105 (92.1)	25.3 (38.8)	0.203^*^
Prenatal antibiotics			
Yes	8 (7.0)	18.4 (23.8)	
No	106 (93.0)	25.3 (38.3)	0.059^*^
Postnatal antibiotics			
Initial, empiric	75 (65.8)	19.0 (33.9)	
Broad-spectrum and prolonged	39 (34.2)	30.0 (45.6)	**0.040** ^*^
Positive blood culture			
Yes	8 (7.1)	21.8 (33.5)	
No	104 (92.9)	23.4 (38.4)	0.842^*^
PROM			
Yes	18 (15.8)	21.6 (28.5)	
No	96 (84.2)	23.4 (40.0)	0.361^*^
Prenatal progesterone			
Yes	19 (16.7)	29.6 (27.1)	
No	95 (83.3)	21.5 (38.8)	0.450^*^
Prenatal dexamethasone			
Yes	26 (23.6)	25.6 (20.9)	
No	84 (76.4)	20.2 (38.4)	0.725^*^
Methyldopa (mother)			
Yes	16 (14.5)	24.3 (40.1)	
No	94 (85.5)	22.2 (32.5)	0.794^*^
Anemia (mother)			
Yes	8 (7.2)	28.3 (42.5)	
No	103 (92.8)	21.5 (34.3)	0.468^*^
Thrombophilia (mother)			
Yes	13 (11.7)	23.4 (27.7)	
No	98 (88.3)	22.2 (38.8)	0.511^*^
Mortality			
Yes	4 (3.5)	35.7 (47.8)	
No	110 (96.5)	23.4 (36.6)	0.877^*^
NEC			
Yes	8 (7.0)	29.2 (60.1)	
No	106 (93.0)	22.2 (37.1)	0.463^*^
Significant ductus arteriosus			
Yes	12 (19.0)	60.8 (92.5)	
No	51 (81.0)	25.0 (34.4)	**0.025** ^*^

RDS-Respiratory distress syndrome; PROM-Premature rupture of fetal membranes; NEC-Necrotizing enterocolitis; *Mann–Whitney *U* test; ^**^ Spearman correlation.

Results obtained have shown that the values of f-calprotectin are in a positive correlation with bicarbonates (Table 2), while pH, base excess, partial pressure of oxygen (pO_2_), and carbon dioxide (pCO_2_) analyzed in a sample of capillary arterial blood were not. Significantly higher fecal calprotectin were detected in female newborn (*p* = 0.003) significant ductus arteriosus (*p* = 0.025), postnatal prolonged use of broad-spectrum antibiotics (*p* = 0.040), and vomiting (*p* = 0.038) (Tables 1 and 3).

Significantly higher fecal calprotectin levels (median 35.9; IQR 70.3 μg/g) were detected in subject with feeding intolerance (performed due to vomiting) compared with the group without enteral feeding intolerance (median 20.2; IQR 30.2 μg/g; *p* = 0.038).

Maternal diseases, prenatal use of antibiotics, corticosteroids and other prenatal therapy did not significantly affect f-calprotectin values. Moreover, postnatal age and other clinical characteristics of subjects: RDS, use of conventional mechanical ventilation or inotropes, a complete blood count, C-reactive protein (CRP), serum procalcitonin, urea, creatinine, and positive blood culture did not significantly affect its values (Table 3).

## 4 Discussion

The gastrointestinal tract (GIT) of the newborns is considered sterile at birth. After birth, it becomes rapidly colonized by microorganisms from the birth canal or from the environment. According to the recent hypothesis, the GIT of the neonate is first inhabited by the microorganisms from the mother’s GIT and uterus, followed by the colonization of the bacteria present in mother’s milk. The mechanism is still unclear. Bacterial colonization of the GIT of the newborn can be considered a kind of the fetus’ immune response to adapt to life outside the uterus [20]. Today, it is known that the effect of interaction between intestinal bacteria and fetal development can have long-term consequences, including the development of gastrointestinal, allergic and metabolic diseases.

During the study period, significantly higher fecal calprotectin levels were recorded in female newborns with intrauterine growth restriction (IUGR) than in the group of males without IUGR. Certain studies [21,22,23] revealed a correlation between IUGR, female infants, and adverse perinatal outcomes that include perinatal asphyxia and polycythemia, which may favor the later development of intestinal distress or necrotizing enterocolitis, with a consequent increase in f-calprotectin. However, certain authors [24,25,26] suggest that high levels of f-calprotectin are associated with enteral feeding and do not always imply pathological GIT inflammation in very low body weight (VLBW) infants. New studies and further investigations are needed to determine the mechanisms underlying this.

In the era of personalized medicine, non-invasive biomarkers can play a key role in reducing neonatal mortality, first and foremost, by enabling more accurate assessment of the risk for the development of neonatal diseases, more appropriate therapeutic treatment, and earlier prediction of the clinical outcome. Previous research suggested that the determination of the calprotectin in the stool of preterm infants could be crucially important since it has already been validated in adults and children as a sensitive marker of inflammatory bowel disease (Crohn’s disease and ulcerative colitis [27]. F-calprotectin is still not sufficiently validated as a non-invasive marker of necrotizing enterocolitis, especially for the early stages according to Bell’s criteria. The influence of other factors on the excretion of f-calprotectin in newborns is still incompletely known and controversial [15,26]. The level of f-calprotectin in children born after a cesarean section did not differ significantly in relation to children born after vaginal delivery, and no significant difference was found in its values with respect to gestational age, postnatal age, and birth weight, which is similar to the findings of other authors [1,2].

Due to large inter- and intra-individual variations, the precise determination of the limit values ​​for f-calprotectin remains an unachievable target, and the proposed limit values, depending on the author, range from 200 to 2,000 μg/g [20,21,22]. During our study, the median calprotectin values were 76.12 ± 175.44 μg/g (from a minimum of 4.4 to a maximum of 1,000 μg/g).

Vomiting, compensatory elevated values of bicarbonates, hemodynamically significant ductus arteriosus, and prolonged postnatal treatment of broad-spectrum antibiotics were associated with higher values of fecal calprotectin, as a sign of intestinal distress or GIT inflammation [27,28]. It is known that, in addition to immaturity, inflammation stimulates cyclooxygenase 2 activation and prostaglandin synthesis, which favors the persistence of the ductus arteriosus. On the other hand, its persistence may worsen pre-existing intestinal ischemia [29].

Enteral feeding intolerance is very common in preterm infants [25,28] and can be a sign of reduced gastrointestinal motility due to the insufficiency of insufficient mature enzymatic system (lactase, lipase, enterokinase, etc.) or may be the initial manifestation of the necrotizing enterocolitis (NEC) [30,31,32,33,34,35,36]. In our study, initial empiric antimicrobial therapy (in which the aminoglycosides have been used in conjunction with beta-lactam antibiotics) was initiated promptly at a high risk of sepsis in symptomatic infants. However, the replacement of the empiric therapy, especially with broad-spectrum antibiotics treatment in the setting of negative cultures, correlated with an increase in the values ​​of f-calprotectin in preterm infants. The benefits of initial antibiotic therapy when indicated are clearly enormous, but the continued use of antibiotics without any microbiological justification is dangerous [37] and may lead to gut microbiota imbalance, antibiotic resistance, or the development of NEC with high mortality.

NEC almost exclusively occurs in preterm infants followed by the development of intestinal inflammation with a significant participation of neutrophils, because of the immature enterocyte response to bacterial stimulation and the onset of oral feeding. While some authors find that f-calprotectin levels increase significantly in VLBW premature infants with NEC [30], other authors believe that this parameter does not play a role in the diagnosis of NEC, especially in the early stages of this disease [26]. In our study, the increase in f-calprotectin did not precede the clinical symptoms and radiographic evidence of NEC IIa-IIb stages, according to Bell, in 8 VLBW premature infants (7%). In many published cases, increased f-calprotectin levels occurred later than acute NEC, so sequential measurement of calprotectin values ​​in stool is necessary when diagnosing NEC. This represents one of the limitations of this study. It may be that the limits in the diagnostic value of f-calprotectin come from variables that affect its levels (e.g., postnatal use of antibiotics and/or probiotics, age, etc.) [36]. Certain variables can be the cause of the unusually low values of calprotectin even in cases of fulminant NEC. Despite few reported cases, the correlation of f-calprotectin with acute NEC is important for neonatologists, since tracking the dynamics of its level changes could be useful for the prospective assessment of NEC and the prediction of outcome. NEC was indicated by the bland necrosis observed in respected specimens. The inflammation, if present, is at the junction of the ischemic bowel that maintains the circulation. Calprotectin may then only indicate the infant’s response to an injury that has already occurred. It has recently been reported that the concentration of f-calprotectin was higher in VLBW premature infants with gastrointestinal “injuries” or gut inflammation than in those with a lower degree of systemic inflammation or perinatal stress, similar to our results. This indicates that calprotectin decreases with healing [9,26]. Longitudinal studies in terms of the long-term follow-up of f-calprotectin during the neonatal period could be of crucial importance.

## 5 Conclusion

New findings on calprotectin as a potentially useful non-invasive parameter of the intestinal distress in preterm infants can be of great importance in the era of a personalized medical approach to the newborns. The diagnostic accuracy of f-calprotectin measurement in preterm infants can be confirmed and increased by including this parameter in the panel with other biomarkers, as well as with the better understanding of the factors that affect its excretion. Our results show that an increase in f-calprotectin, in a population of preterm infants, correlates with IUGR, the enteral feeding intolerance, significant patent ductus arteriosus, and prolonged antibiotic therapy. Measurement of f-calprotectin in the first 7 days after birth can help to detect early the intestinal distress or early staging of NEC in premature infants.
